# Violacein Treatment Modulates Acute and Chronic Inflammation through the Suppression of Cytokine Production and Induction of Regulatory T Cells

**DOI:** 10.1371/journal.pone.0125409

**Published:** 2015-05-04

**Authors:** Liana Verinaud, Stefanie Costa Pinto Lopes, Isabel Cristina Naranjo Prado, Fábio Zanucoli, Thiago Alves da Costa, Rosária Di Gangi, Luidy Kazuo Issayama, Ana Carolina Carvalho, Amanda Pires Bonfanti, Guilherme Francio Niederauer, Nelson Duran, Fábio Trindade Maranhão Costa, Alexandre Leite Rodrigues Oliveira, Maria Alice da Cruz Höfling, Dagmar Ruth Stach Machado, Rodolfo Thomé

**Affiliations:** 1 Department of Structural and Functional Biology, Institute of Biology, University of Campinas, Campinas, SP, Brazil; 2 Department of Genetics Evolution and Bioagents, Institute of Biology, University of Campinas, Campinas, SP, Brazil; 3 Biological Chemistry Laboratory, Chemistry Institute, University of Campinas, Campinas, SP, Brazil; 4 Laboratory of Nanostructured Synthesis and Biosystems Interactions (NanoBioss), Chemistry Institute, University of Campinas, Campinas, SP, Brazil; 5 Department of Histology and Embryology, Institute of Biology, University of Campinas, Campinas, SP, Brazil; University of Tokyo, JAPAN

## Abstract

Inflammation is a necessary process to control infection. However, exacerbated inflammation, acute or chronic, promotes deleterious effects in the organism. Violacein (viola), a quorum sensing metabolite from the Gram-negative bacterium *Chromobacterium violaceum*, has been shown to protect mice from malaria and to have beneficial effects on tumors. However, it is not known whether this drug possesses anti-inflammatory activity. In this study, we investigated whether viola administration is able to reduce acute and chronic autoimmune inflammation. For that purpose, C57BL/6 mice were intraperitoneally injected with 1 μg of LPS and were treated with viola (3.5mg/kg) via i.p. at the same time-point. Three hours later, the levels of inflammatory cytokines in the sera and phenotypical characterization of leukocytes were determined. Mice treated with viola presented a significant reduction in the production of inflammatory cytokines compared with untreated mice. Interestingly, although viola is a compound derived from bacteria, it did not induce inflammation upon administration to naïve mice. To test whether viola would protect mice from an autoimmune inflammation, Experimental Autoimmune Encephalomyelitis (EAE)-inflicted mice were given viola i.p. at disease onset, at the 10th day from immunization. Viola-treated mice developed mild EAE disease in contrast with placebo-treated mice. The frequencies of dendritic cells and macrophages were unaltered in EAE mice treated with viola. However, the sole administration of viola augmented the levels of splenic regulatory T cells (CD4+Foxp3+). We also found that adoptive transfer of viola-elicited regulatory T cells significantly reduced EAE. Our study shows, for the first time, that violacein is able to modulate acute and chronic inflammation. Amelioration relied in suppression of cytokine production (in acute inflammation) and stimulation of regulatory T cells (in chronic inflammation). New studies must be conducted in order to assess the possible use of viola in therapeutic approaches in human autoimmune diseases.

## Introduction

Violacein (viola) is a compound derived from bacterium *Chromobacterium violaceum* and acts as a quorum sensing metabolite that also possess anti-microbial, anti-tumor and anti-malarial activity [1–3]. It was previously shown that viola treatment results in elimination of *Plasmodium falciparum* parasites within erythrocytes [4]. In addition, administration of viola inhibits tumor growth [5–7] In this context, it is proposed that viola acts through inhibition of cell growth and induction of apoptosis [5, 6]. Although the mechanism of action has not been fully characterized; collectively, these findings suggest that viola may present a broad range of action [1, 4–9]. Notwithstanding, it is imperative to discover new therapeutical agents in the treatment of inflammatory conditions. In this sense, diseases, especially autoimmune-driven ones, such as human multiple sclerosis and its animal model—Experimental Autoimmune Encephalomyelitis (EAE), lack new anti-inflammatory drugs.

The mechanisms that trigger the development of Multiple Sclerosis are not fully understood. However, it is well-established that EAE develops after encephalitogenic T cells that have been activated in the periphery of the immune system and then extensively proliferated and migrated to the Central Nervous System (CNS), where they secrete inflammatory cytokines, enzymes and chemo-attractive molecules [10]. The local inflammation in the CNS is a problem normally bypassed after treatment with anti-inflammatory drugs. As the modulation of the immune system is a necessary step to achieve efficient treatments for chronic inflammatory diseases, several new drugs are being tested in animal models of autoimmune diseases aiming their future use in the clinic. Drugs such as chloroquine, dihidroartemisinin, and primaquine have already been used to modulate EAE and experimental arthritis [11–14] and some of them are currently in use in the clinic [15]. Still, other compounds must be characterized for future treatment design.

In this context, we aimed to evaluate whether the treatment with viola would modulate acute inflammation as well as the clinical course of EAE. We found that administration of viola was able to reduce systemic inflammation induced with LPS administration. In addition, our results show that upon viola administration the EAE severity was significantly reduced in comparison with the placebo-treated group. In these viola-treated mice, CNS expression of inflammatory cytokine was abolished. Viola treatment also enhances the frequency of regulatory T (Treg) cells and the adoptive transfer of viola-elicited Treg cells was able to mimic the results observed with the full compound administration. To our knowledge, this is the first study to show that the bacterial compound viola is able to suppress EAE through the inhibition of local inflammation and induction of Treg cells.

## Materials and Methods

### Animals

Female C57BL/6 mice (n = 6, 6–8 week-old) were used in this study. Mice were obtained from the University of Campinas Center for animal care (CEMIB). All mice were specific-pathogen free and acclimatized in clean cages with autoclaved food and water *ad libitum*. The photoperiod was controlled (12h/12h, light/dark) as well as the temperature (22°C). Before experimental procedures that required killing of animals, mice were euthanized under deep anesthesia (intraperitoneal injection of 160 mg/kg of ketamine and 30 mg/kg of xylazine). All protocols involving laboratory animals were approved and performed in accordance with the guidelines of the University of Campinas’ Committee on the Use and Care of Animals (CEUA) under protocol number 2687–1.

### Violacein (viola) production.

Viola was extracted from *Chromobacterium violaceum* as previously described [1, 4]. Briefly, violacein (3—(1,2-dihydro- 5-(5-hydroxy- 1H -indol-3-yl) -2-oxo -3H- pyrrol—3ylidene) -1,3- dihydro -2H -indol-2-one) was extracted through purification with chloroform, followed by diethyl ether and then ethanol. After evaporation of ethanol, the viola was purified by crystallization (methanol/water) followed by HPLC (High Performance Liquid Chromatography). DMSO was used to dissolve viola due to its poor solubility in water. Lipopolysaccharide residues were not detected (using the Limulus Amebocyte Lysate QCL -1000 kit, Cambrex, USA).

To verify drug toxicity, mice were intraperitoneally treated with viola at the doses 1.75; 3.5 and 7 mg/Kg. The treatment regimen consisted in daily intraperitoneal (i.p.) administration of viola for three consecutive days. Mice receiving the 7mg/Kg dose died after the second dose.

### Acute inflammation model and viola treatment.

The acute inflammation model was induced by the administration of lipopolysaccharide (1μg/mouse, from E. coli 0111:B4, Sigma-Aldrich, USA) via i.p. as previously described [16]. Violacein (3.5 mg/kg) was administrated at the same time-point through the i.p. route. Three hours after the induction of inflammation, mice were killed and sera and spleens were collected. Cytokine in sera levels were determined by Cytometric Bead Array (CBA, The Mouse Inflammation Kit, BD Bioscieces, USA). Spleens were cut into small pieces and disrupted to prepare single-cell suspensions. The cells were surface stained with fluorochrome-conjugated monoclonal antibodies against CD11b (M1/70), Ly6G (RB6-8C5), CD19 (MB19-1), F4/80 (BM8), CD11c (N418), CD80 (16-10A1) and CD86 (GL1), CD3 (145-2C11), CD4 (GK1.5), CD8α (53–6.7) and CCR7 (4B12) on ice for 30 minutes. Antibodies were from BD Biosciences (USA). Later, events were acquired on Gallios Flow Cytometer (Becman Coulter, USA) and analyzed in FlowJo VX (FlowJo Inc., USA).

### Experimental Autoimmune Encephalomyelitis (EAE) induction.

EAE was induced and evaluated in mice according to a previous published paper [17]. Briefly, mouse was injected with MOG_35–55_ peptide (100μg, Genescript, USA) emulsified in Complete Freund´s Adjuvant (CFA, Sigma-Aldrich, USA). Pertussis toxin (200ηg, Sigma-Aldrich, USA) was administrated via i.p. at 0 and 48h after MOG immunization. The maximum tolerable dosage (3.5mg/kg) of viola was used to treat EAE-bearing mice. The treatment started at disease onset (around the 10^th^ day after MOG immunization) and continued for three consecutive days. As control, half of the naïve mice were treated with viola (viola group) while EAE-bearing mice received vehicle (referred to as EAE group). Clinical signs of the disease were followed and graded daily according to a score method, where: 0-no sign, 1-limp tail, 2-hind limbs weakness, 3-hind limb paralysis, 4-hind paralysis and fore limbs weakness, 5-full paralysis/death.

### Analysis of the frequency of F4/80^+^ and CD11c^+^MHC-II^+^ cells in the spleens of EAE-bearing mice.

At the 20^th^ day after EAE induction, mice were killed and the spleens were collected and fragmented into small pieces. Later, the organs were disrupted to prepare single cell suspensions which were surface stained with fluorochrome-conjugated monoclonal antibodies against F4/80 (clone BM8), CD11c (clone N418), CD80 (clone 16-10A1) and CD86 (clone GL1) for 30 min on ice. Antibodies were from BD Biosciences (USA). Preparations were acquired in Gallios Flow Cytometer (Becman Coulter, USA) and analyzed in FlowJo VX (FlowJo Inc., USA).

### Analysis of the gene expression in CNS.

At the 20^th^ day after EAE induction, mice were killed and the lumbar spinal cords were removed and frozen immediately. The frozen tissues of CNS were used for RNA extraction using Trizol (Life Technologies, USA) and cDNA synthesis was performed according to the manufacturer´s recommendations (High Capacity c DNA Converter Kit, Life Technologies, USA). FOXP3 (Mm00475162_m1), *Indoleamine 2*,*3-Dioxygenase* (IDO) (Mm00492586_m1), *Inducible Nitric Oxide Synthase* (iNOS) (Mm00440502_m1), IL-17 (Mm00439618_m1), IL-10 (Mm00439614_m1), TGF-β (Mm00498255_m1), IFN-γ (Mm01168134_m1) and TNF-α (Mm00443258_m1) were analyzed in comparison to GAPDH (Mm99999915_g1, housekeeping gene) levels. RT-PCR reactions were performed using Taqman reagents according to manufacturer´s recommendations (Applied Biosystems, USA). The results are shown as relative expression (2^-ΔΔCT^).

### Analysis of cellular infiltration in the CNS.

At the 20^th^ day after EAE induction, mice were anesthetized, perfused with ice cold PBS and the lumbar spinal cords were removed and prepared for the enrichment of infiltrating leukocytes according to a previously described methodology [18, 19]. Briefly, spinal cord-derived cells were suspended in Percoll 30%, and then overlaid in Percoll 70%. The suspension was centrifuged for 30 minutes at 320g and no breaks. The cells in the interface were collected and washed in Phosphate-Buffered Saline (PBS, 0,02M pH 7.2) and counted in hemocytometer.

### Analysis of the frequency of regulatory T cells and intracellular cytokine detection after viola treatment.

Naïve C57BL/6 mice were treated with viola as above and three days after the last dose of viola, mice were killed and splenocytes were collected. Single cell suspensions were prepared and surface stained with fluorochrome-conjugated antibodies against: anti-CD4/PE-Cy5 (clone GK1.5), anti-CD3/APC-Cy7 (clone 145-2C11), anti-CTLA-4/PE (clone UC10-4B9) and anti-CD25/FITC (clone PC61). For intracellular staining of Foxp3, the cells were treated with “fixation/permeabilization” buffers according to the manufacturer´s recommendations (eBioscience, USA). Anti-FOXP3/APC (Clone FJK-16s) antibodies were added to the cells. For intracellular detection of cytokines, spleen cells were stimulated with Phorbol 12-myristate 13-acetate (PMA, 50 ng/mL, Sigma-Aldrich, USA) and Ionomycin (500 ng/mL, Sigma-Aldrich) in the presence of Brefeldin A (1 μg/mL, Sigma-Aldrich) for 4h at 37°C. Later, cells were surface stained with anti-CD4/PE-Cy5 (clone GK1.5) for 20 minutes at 4°C and fixed and permeabilized with Fixation/Permeabilization Buffer (eBioscience, USA). Antibodies against IL-10/APC (clone JES5-16E3), IFN-γ/PE (clone XMG1.2) and IL-17/FITC (clone eBIO17B7) were added and the suspensions were incubated at 4° for 30 minutes. All antibodies were from eBiosciences. Preparations (at least 50.000 events) were acquired in a Gallios Flow Cytometer (Becman Coulter, USA) and data analyzed using FlowJo 7.6 software (Tree Star Inc., USA).

### Isolation of Tregs (CD4^+^CD25^+^), adoptive transfer experiments and suppressive assays.

Naïve C57BL/6 mice were treated with viola as described above and at the end of the treatment period spleen cells were collected and CD4^+^CD25^+^ cells were isolated using magnetic beads following manufacturer´s instruction (Mouse Regulatory T Cell Dynabeads, Life Technologies, USA). For adoptive transfer experiments, 5x10^5^ Tregs per mouse were adoptively transferred (by the venous route) to EAE-inflicted mice at the onset of the disease (10 days after MOG immunization). As controls, EAE mice received equal numbers of CD4^+^CD25^-^ cells (effector T cells, Teff) at the same time-point. EAE induction and evaluation was performed as described above. To evaluate the suppressive function of Treg cells from viola-treated mice, Treg cells were isolated from naïve and viola-treated mice and seeded at increasing numbers in 96 U-bottom wells culture plates. Later, spleen T cells from MOG_35–55_-immunized mice (seven days after immunization) were isolated with FlowComp Mouse T cells Dynabeads (LifeTechnologies) according to manufacturer’s recommendations. Encephalitogenic T cells were stained with CFSE (1.5 μM, Sigma-Aldrich) for 5 minutes in the dark in PBS. Stained cells were washed with RPMI medium supplemented with Fetal Calf Serum (10% v:v, Cultilab, BRA) to block dye activity and then were seeded (5x10^5^ cells/well) to the plates containing unstained isolated Treg cells. Cells were cultured in the presence of DCs (5x10^4^/well) from MOG_35–55_-immunized mice. As controls, CFSE-stained encephalitogenic T cells were cultivated with DCs but without Treg cells. Cultures were incubated for 72h at 37°C. After the culture period, cells were collected, fixed in formaldehyde 4% and acquired in flow cytometer (Gallios, Becman Coulter, USA). The frequency of CFSE^low^ (proliferating) cells was evaluated in each culture condition. Suppressive activity of Treg cells was determined according to the index of proliferation in co-cultures compared with cultures of encephalitogenic T cells without Treg cells. Alternatively, Treg cells from PBS- and viola-treated mice were seeded in 96-well culture plates at a density of 5x10^5^ cells/well and incubated for 48h at 37°C. After the incubation time, the supernatant was collected and assayed for IL-10 detection with CBA, as described previously.

### Statistical analyses.

Clinical score comparisons between control and experimental groups were carried out by Two-Way ANOVA and post-tested with Bonferroni. Other analyses among two and three (or more) groups were done with Student´s t test and One-Way ANOVA, respectively. Results are expressed as mean ± standard error mean (SEM) and values of p<0,05 were defined as significant.

## Results

### Violacein treatment reduces acute inflammation.

**Fig 1:**
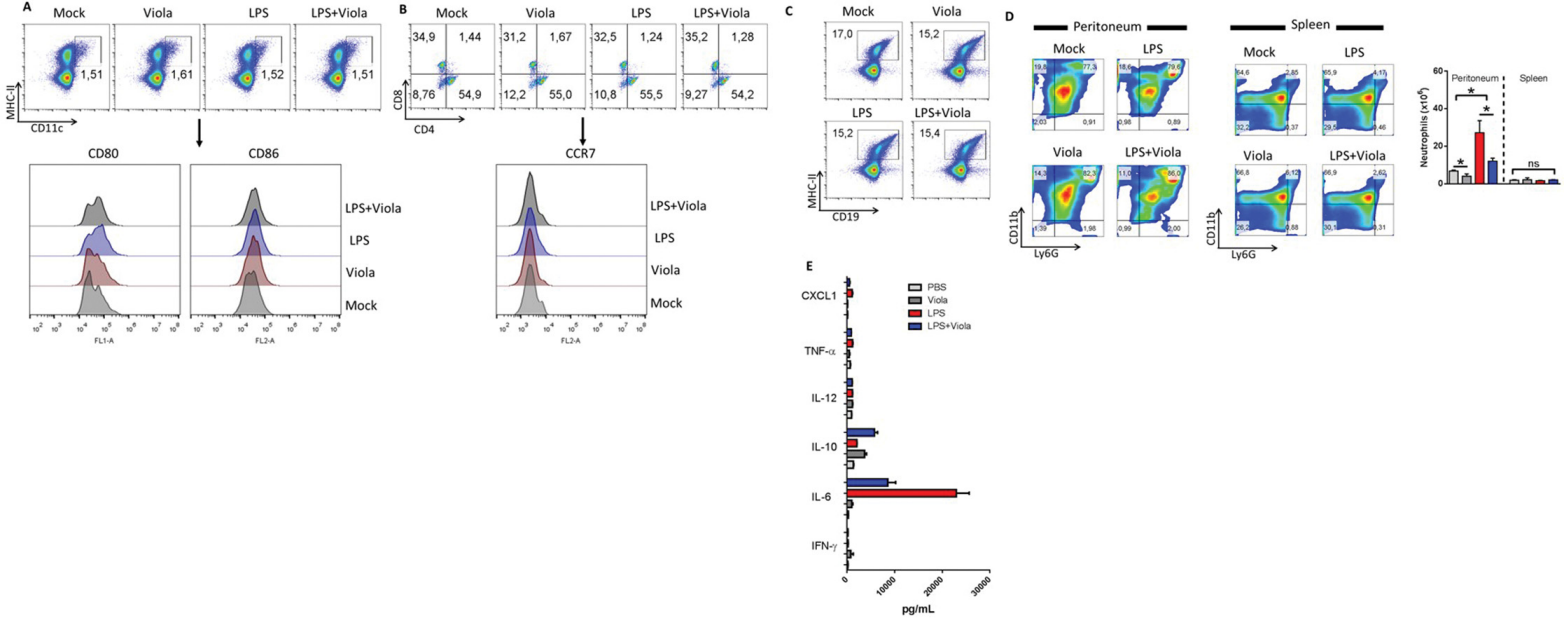
Violacein administration diminishes acute inflammation induced by Lipopolysaccharide injection. C57BL/6 mice (n = 3 mice/group) were treated with viola (3.5 mg/Kg) and lipopolysaccharide (1μg/mouse) through the intraperitoneal route. Three hours after LPS injection, mice were killed and the frequencies of dendritic cells (in A), T lymphocytes (in B), B cells (in C) and Neutrophils (in D) were assessed. E) The serum levels of selected cytokines and CXCL1 were determined as well. Representative data from two independent experiments.

To evaluate whether the treatment with viola is able to suppress acute inflammation, mice were administrated with 1μg of LPS via i.p. A single dose of viola was added at the same time-point. Three hours after LPS injection, mice were killed and the frequency of DCs was evaluated. The results show that in this acute model of inflammation, DCs frequencies did not change as well as the expression of maturation markers CD80 and CD86 inside the DC population from LPS-treated mice (Fig 1A). The frequency of T cells was also analyzed, and results showed that the treatment with viola+LPS did not present toxic effect on these populations nor did it induce the expression of the chemokine receptor CCR7 inside the CD3^+^ T cell population (Fig 1B). Similar results were observed in the frequency of B cells (Fig 1C). Interestingly, although their phenotype did not change, viola-treated mice injected with LPS displayed a significant reduction in the infiltration of neutrophils to the peritoneal cavity compared with controls (Fig 1D). This effect on neutrophils numbers was not observed in the spleens of mice. On the other hand, the serum levels of the inflammatory cytokine IL6 and CXCL1 were reduced in mice treated with viola whereas the levels of the anti-inflammatory cytokine IL-10 were augmented (Fig 1D). These results show that viola treatment does not alter the frequency of dendritic cells and lymphocytes, but reduces the migration of neutrophils to the perintoneum instead. Somehow the level of IL-10 was found up regulated whereas IL-6 and CXCL1 were reduced, which suggest that the suppressive effect of a single viola administration relies on cytokine and chemokine modulation.

### Violacein treatment reduces the clinical score of EAE.

**Fig 2:**
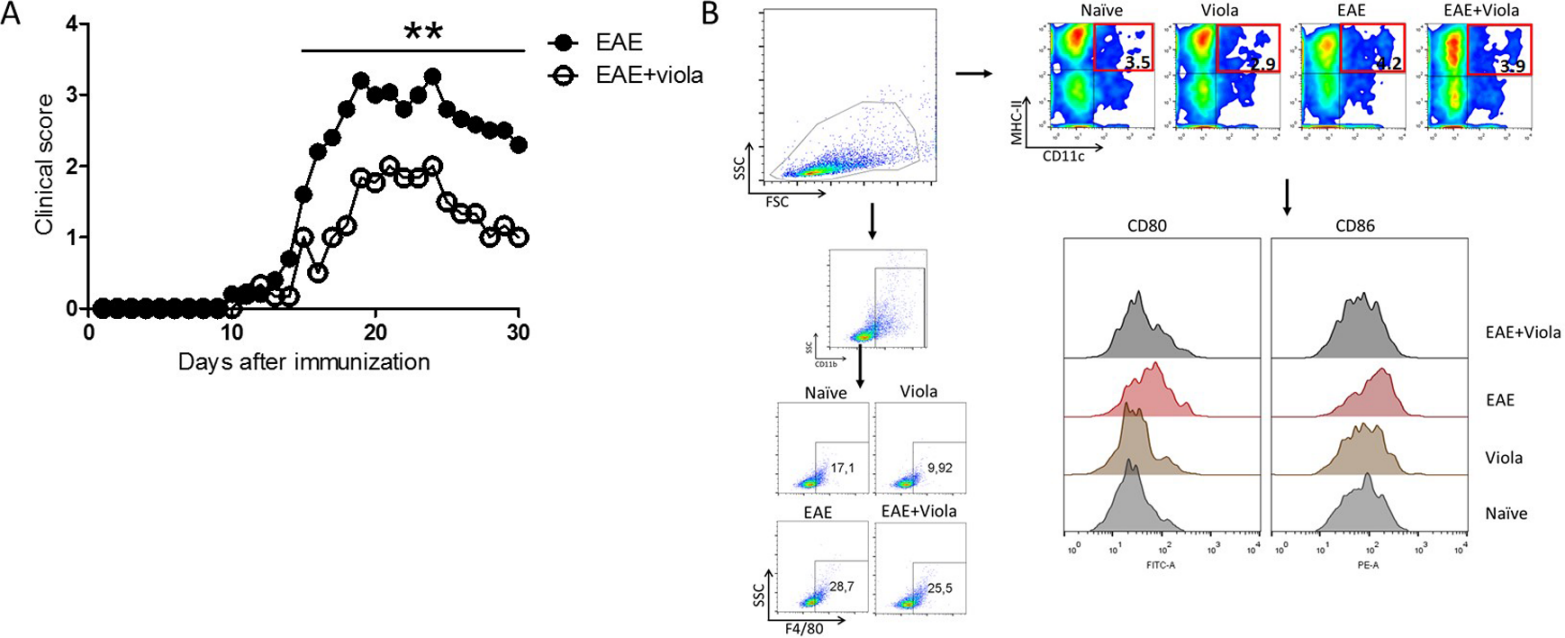
Treatment with violacein reduces the severity of Experimental Autoimmune Encephalomyelitis. C57BL/6 mice (n = 6 mice/group) were immunized with MOG_35–55_ peptide emulsified in Complete Freund’s Adjuvant (CFA) and intraperitoneally injected with Pertussis toxin (200ηg) at 0 and 48h from immunization. Ten days after immunization, when the first clinical signs started to appear, mice were treated with violacein (3.5 mg/Kg) for three consecutive days. A) Results show that the treatment reduced the severity of EAE. B) Later mice were killed and the frequency of macrophages (F4/80+ cells) and dendritic cells (CD11c+MHC-II+ cells) were assessed. The expression of CD80 and CD86 on DCs was evaluated as well. Representative data from three independent experiments. **p<0,01.

In order to investigate whether viola treatment would be able to reduce ongoing chronic inflammation, we immunized mice with encephalitogenic peptide and evaluated the progression of EAE clinical score. Mice developed the EAE first clinical sign (limp tail) at the 10^th^ day after MOG immunization. A group of mice was treated for three days with viola (3.5mg/Kg), the EAE+Viola group. As shown in Fig 2, the treatment with viola reduced the clinical course of EAE. Although mice were treated only for three days, the suppression of the disease persisted until the 30^th^ day after immunization. The treatment triggered a statistically significant improvement in the disease progression (Fig 2A). Likewise, in the untreated EAE group, the peak clinical score reached 4 while in viola-treated mice the maximum score was 2 (Table 1). Disease incidence was altered as well, with lower incidence of the disease among the subjects in EAE+viola group compared with EAE group (Table 1).

**Table 1:** Effect of viola treatment and viola-elicited Treg cells adoptive transfer on the severity of EAE.

Group	Incidence	Maximum score	Day of disease outcome (±SEM)	Mean Clinical Score (±SEM)
Naïve	0/18	0	0	0
Viola	0/18	0	0	0
EAE	18/18	4	10±1,0	2,8±1,0
EAE+Viola	5/12*	2	10±1,0	1,5±0,5*
EAE+CD25^-^	12/12	3	10±1,0	2,5±0,8
EAE+CD25^+^	8/12* ^#^	2	10±1,0	1,3±0,3* ^#^

*: p<0,05, in comparison with the EAE group;

#: p<0,05, in comparison with the EAE+CD25^-^ group.

Analyses were performed with One-Way ANOVA followed by Bonferroni’s posttest.

### Altered activation of dendritic cells from EAE+viola mice.

Following viola treatment, mice were killed and the frequencies of macrophages and dendritic cells (DCs) in the spleens were assessed. The results showed that the sole treatment with viola reduced the frequency of macrophages while in the EAE and EAE+viola groups the frequencies remained similar (Fig 2B). On the other hand, the treatment with viola did not change the frequencies of dendritic cells in the spleens. However, the activation profile of DCs was altered. DCs from the EAE+viola group presented lower expression of CD80 and CD86 compared with the EAE group (Fig 2B).

### Reduced CNS inflammation and cellular infiltration in EAE mice treated with viola.

**Fig 3:**
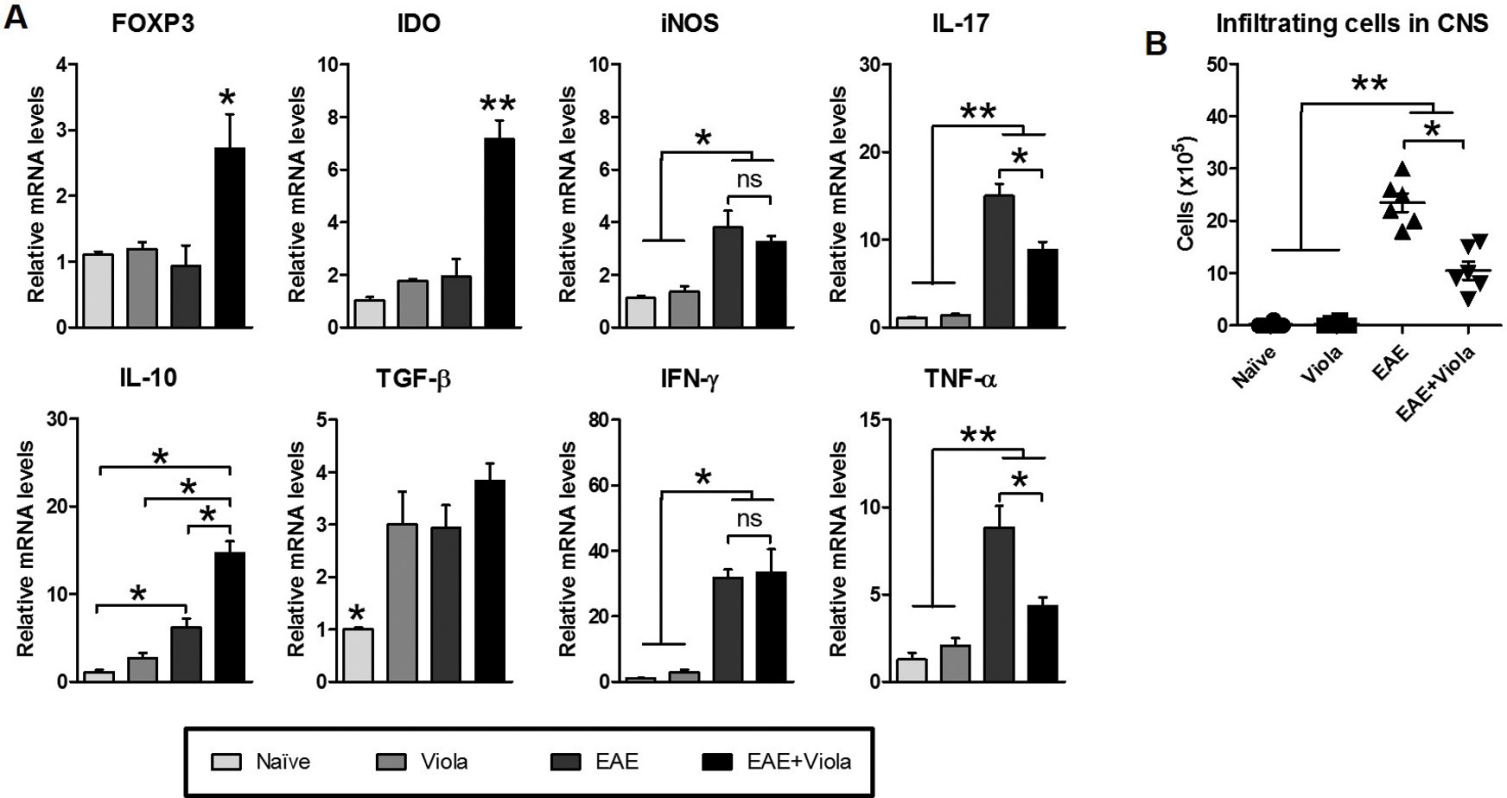
Treatment with violacein reduces the severity of Experimental Autoimmune Encephalomyelitis through the modulation of CNS inflammation and infiltration of cells. C57BL/6 mice (n = 6 mice/group) were immunized with MOG_35–55_ peptide emulsified in Complete Freund’s Adjuvant (CFA) and intraperitoneally injected with Pertussis toxin (200ηg) at 0 and 48h from immunization. Ten days after immunization, when the first clinical signs started to appear, mice were treated with viola (3.5 mg/Kg) for three consecutive days. At the 20^th^ day after immunization, mice were killed and lumbar spinal cords were removed and submitted to mRNA extraction protocols. A) The expression of FOXP3, IDO, iNOS, IL-17, IL-10, TGF-β, IFN-γ and TNF-α was evaluated and show that viola treatment changed the CNS gene expression profile of EAE-bearing mice. B) The infiltration of cells in the CNS was evaluated as well. For that, spinal cords were collected and enriched in T cells, which were counted in hemocytometer. Results show that the treatment reduced the infiltration of cells in the CNS of EAE-bearing mice. Representative data from two independent experiments. All values are represented as means ± standard error mean. *p<0.05 and **p<0.01.

Later, the inflammation in CNS of viola-treated mice was evaluated. At the 20^th^ day after MOG immunization, mice were killed and lumbar spinal cords were collected and proceeded by mRNA extraction. The gene expression of inflammatory mediators and transcription factors were analyzed. PBS-treated mice bearing EAE presented significant higher expression levels of iNOS, IL-17, IFN-γ, TNF-α and TGF-β compared with naïve and viola mice. There was no statistical differences in IFN-γ and TGF-β expression between EAE and EAE+Viola groups. However, in CNS from viola-treated EAE-bearing mice (EAE+viola group), the expression of Foxp3, IDO and IL-10 was significantly higher compared to the EAE control group (Fig 3A). In order to investigate whether the reduction in the expression of inflammatory mediators were related to the infiltration of cells, mice were killed at the same time-point as described previously. The infiltrating cells were enriched in density gradient centrifugation and counted. The results showed that the treatment with viola reduced the number of cells infiltrating the CNS in comparison with untreated EAE mice (Fig 3B).

Taken together, the results presented so far show that EAE reduction correlated with an altered activation/maturation profile of DCs in the spleens and a reduction in the inflammation in the CNS of mice treated with viola.

### Increased numbers of Regulatory T cells in mice treated with viola.

**Fig 4:**
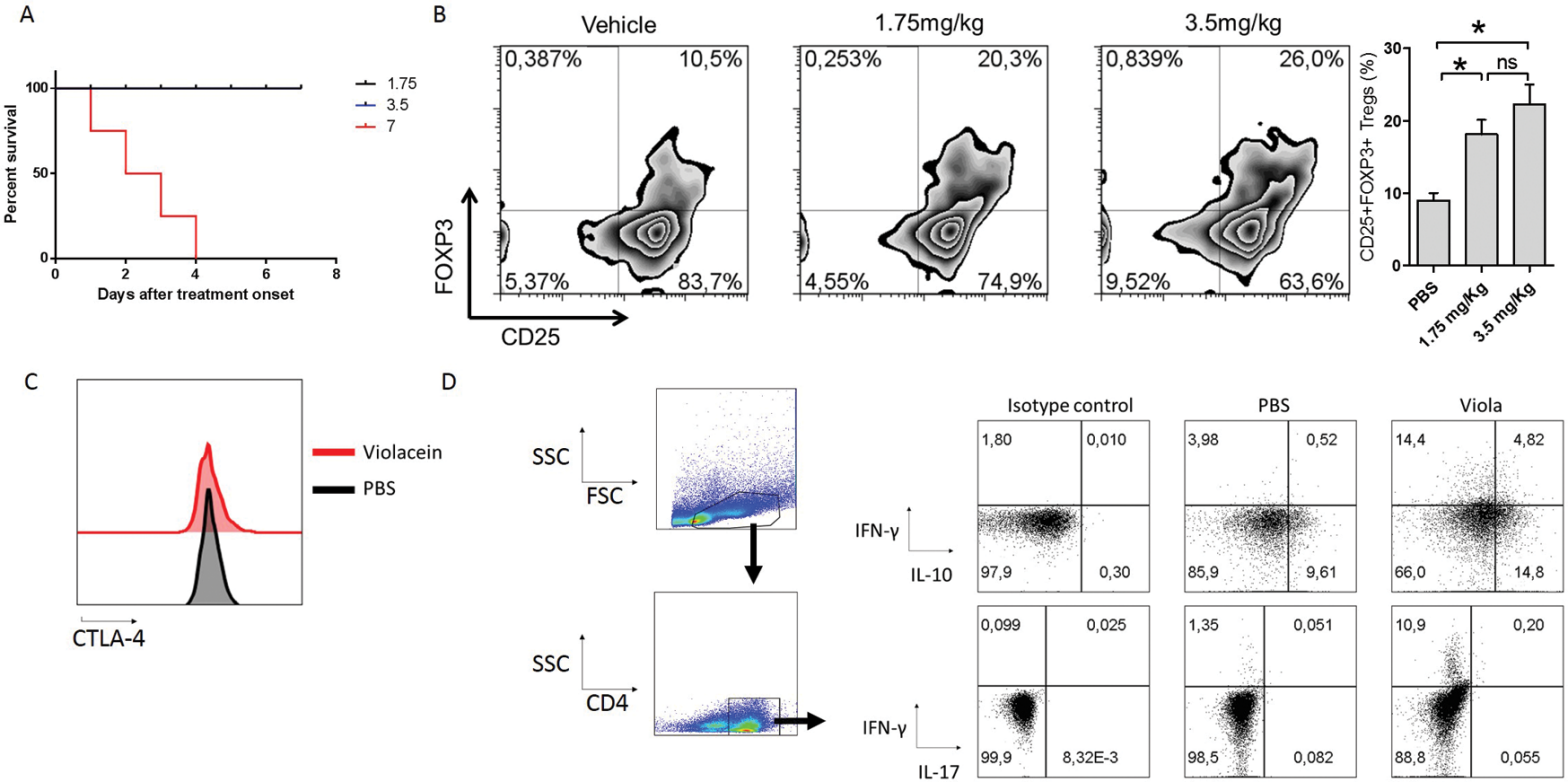
Violacein treatment up-regulates the frequency of Regulatory T cells. C57BL/6 mice (n = 6 mice/group) were treated with viola (1.75, 3.5 and 7 mg/Kg) for three consecutive days. A) Kaplan-Meier curve of survival show that mice receiving the 7mg/Kg dose died after the second administration. B) After the last dose of viola, mice were killed and spleen cells were stained for flow cytometry analysis of CD4^+^CD25^+^FOXP3^+^ (Treg) cells. Results show an increase in the frequency of Treg cells after viola treatment. C) Expression of CTLA-4 was evaluated inside the Treg cell population by flow cytometry. D) The intracellular cytokine detection was performed in spleen cells from PBS- and viola-treated mice. The spleen cells were stimulated with PMA (50 ng/mL) and Ionomycin (500 ng/mL) in the presence of Brefeldin A (1 μg/mL) for 4h at 37°C. Later cells were surface stained with antibody against CD4. Following permeabilization, cells were incubated with antibody cocktail for the detection of IL-17, IL-10 and IFN-γ and preparations were acquired in Flow Cytometer equipment. Representative data from three independent experiments. All values in bar graphs are represented as means ± standard error mean. *p<0.05.

The increased expression of Foxp3, IL-10 and TGF-β in the CNS of viola treated mice and the alterations in the serum of IL-6 and IL-10 following viola treatment suggests that regulatory T (Treg) cells are involved in the suppression of inflammation. To assess this possibility, naïve mice were treated with viola at the doses 1.75, 3.5 and 7 mg/Kg. Mice that received the 7mg/Kg dose died shortly after the second dose (Fig 4A). After the administration of the last dose of viola, mice were killed and the splenic levels of CD4^+^CD25^+^FOXP3^+^ (regulatory T cells) were assessed. The data obtained showed that the treatment with viola promotes a significant up-regulation in the frequency of Treg cells when compared with vehicle-treated mice (Fig 4B). There was no statistical difference between mice that received 1.75 and 3.5 mg/Kg dose. Further investigation revealed that there was no statistical difference in the expression of CTLA-4 between Treg cells from PBS and viola-treated mice as well (Fig 4C). In order to evaluate whether violacein treatment alters the pattern of cytokine production by T cells, we treated mice with viola as described above and collected the spleen cells. After stimulation with PMA+Ionomycin in the presence of brefeldin A, intracellular levels of IFN-γ, IL-17 and IL-10 were analyzed inside the CD4^+^ population. We observed that viola treatment significantly augmented the production of IL-10 and IFN-γ by CD4^+^ T cells, whereas had no influence in the production of IL-17 (Fig 4D).

### Adoptive transfer of violacein-elicited Regulatory T cells reduces the clinical course of EAE.

**Fig 5:**
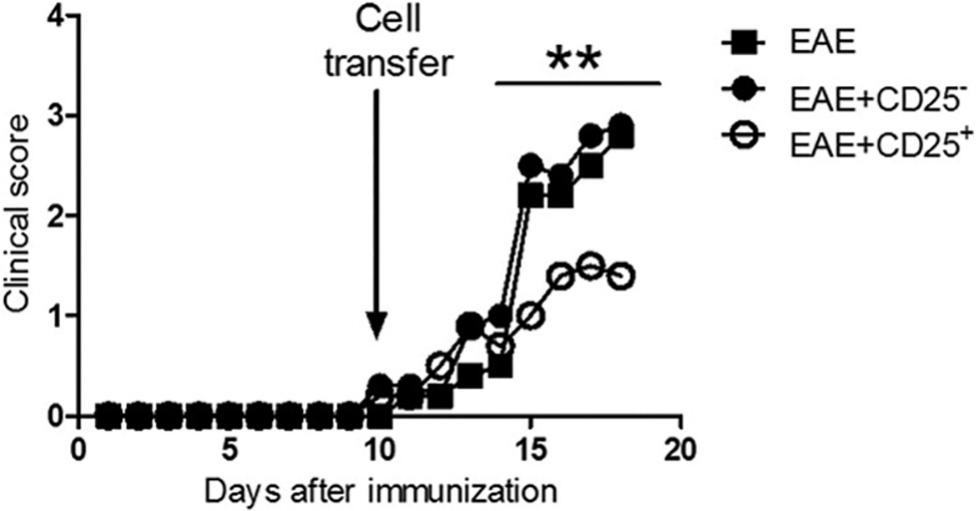
Reduction in EAE severity after adoptive transfer of violacein-elicited Treg cells. C57BL/6 mice (n = 6 mice/group) were treated with viola (3.5mg/Kg) for three consecutive days. After the last dose of viola, mice were killed and spleen cells were prepared for single cell suspension. CD4^+^CD25^+^ and CD4^+^CD25^-^ cells were isolated using dynabeads, following manufacturer’s instructions (Life Technologies). 5x10^5^ cells were adoptively transferred into EAE-bearing mice, at the 10^th^ day after immunization (arrow), the clinical course was evaluated daily and showed reduction in the severity of CD4^+^CD25^+^-transferred mice compared to the other groups. Representative data from two independent experiments. All values are represented as means from each group. **p<0.01.

To investigate whether viola-elicited Treg cells are responsible for the suppression of EAE, naïve mice were treated with viola as described previously. At the end of the treatment, mice were killed and spleen Treg cells were isolated and adoptively transferred towards EAE-bearing mice (at the 10^th^ day after MOG immunization). As demonstrated in Fig 5, the transfer of Treg cells significantly reduced the severity of EAE when compared with control EAE mice and effector T cell (Teff, CD25^-^)-transferred EAE mice. Interestingly, the single transfer of regulatory T cells was able to produce a persistent amelioration of the disease (Fig 5). Mice recipient of Treg cells developed a mild EAE clinical score with maximum score of 2, whereas mice from the EAE+CD25^-^ group presented a maximum score of 4 (Table 1).

### Reduced EAE clinical course correlates with reduction in gene expression of inflammatory cytokine within the CNS.

**Fig 6:**
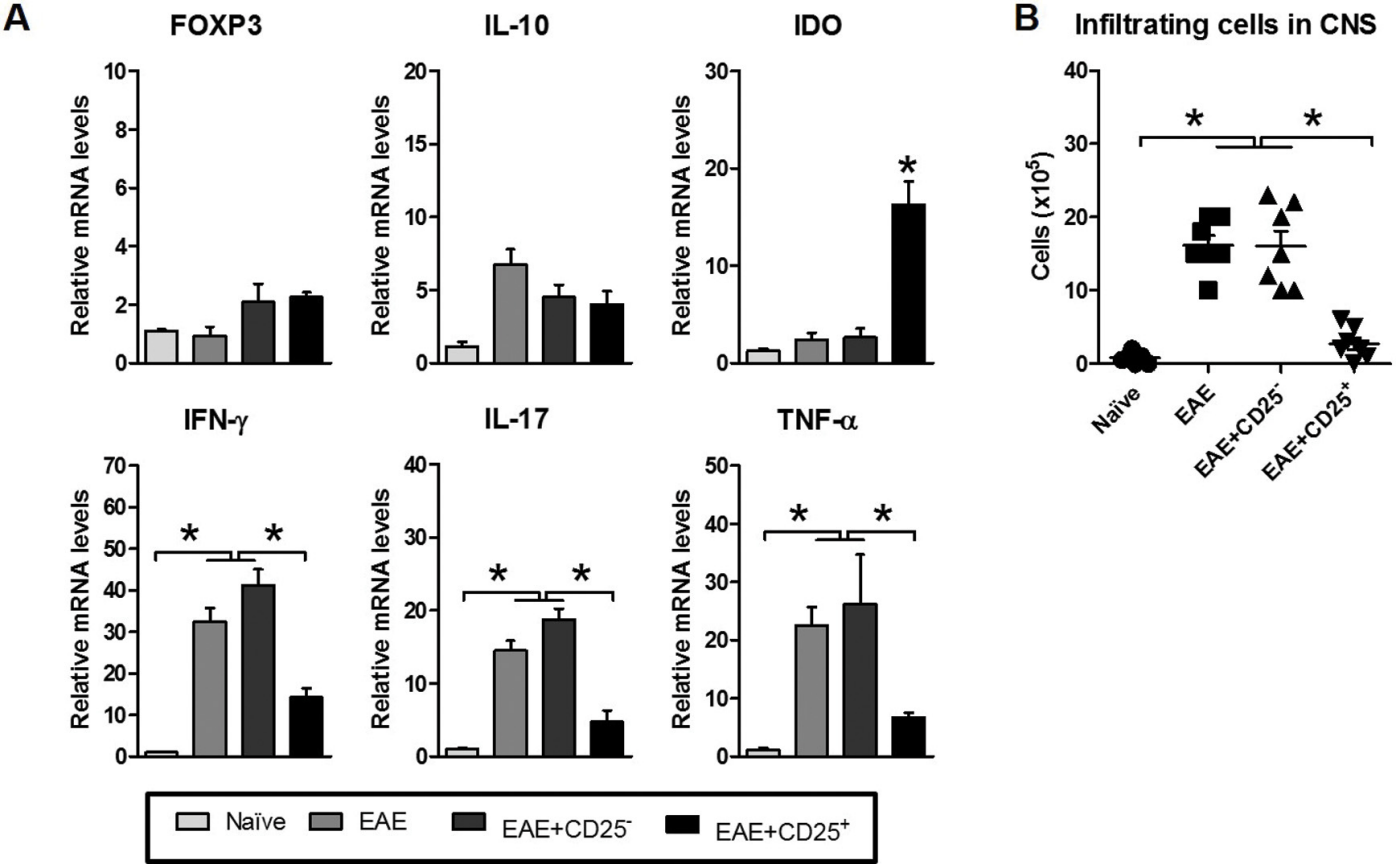
Adoptive transfer of regulatory T cells elicited after violacein treatment reduces EAE through the modulation of CNS inflammation and inhibition infiltration of cells. C57BL/6 mice (n = 6 mice/group) were treated with viola (3.5mg/Kg) for three consecutive days. After the last dose of viola, mice were killed and spleen cells were prepared for single cell suspension. CD4^+^CD25^+^ and CD4^+^CD25^-^ cells were isolated using dynabeads, following manufacturer’s instructions (Life Technologies). 5x10^5^ cells were adoptively transferred into EAE-bearing mice, at the 10^th^ day after immunization, the clinical course was evaluated daily. At the 20^th^ day after immunization, mice were killed and lumbar spinal cords were removed and submitted to mRNA extraction protocols. A) The expression of FOXP3, IDO, IL-17, IL-10, IFN-γ and TNF-α was evaluated. Results show that adoptive transfer of viola-elicited Treg cells changed the CNS gene expression profile of EAE-bearing mice. B) The infiltration of cells in the CNS was evaluated as well. For that, spinal cords were collected and enriched in T cells, which were counted in hemocytometer. Results show that the treatment inhibited the infiltration of cells in the CNS of EAE-bearing mice. Representative data from two independent experiments. All values are represented as means ± standard error mean. *p<0.05.

The gene expression levels in the CNS of EAE-bearing mice recipient of Teff and Treg cells were analyzed and compared to the levels of EAE control mice. The results show that mice recipient of Treg cells presented significant higher levels of IDO expression when compared with the other groups (Fig 6A). Levels of IFN-γ, IL-17 and TNF-α were found down regulated in comparison with CD25^−^-recipient mice and EAE control group. Interestingly, levels of FOXP3 and IL-10 were not statistically different among groups (Fig 6A). To assess if these results are related to the infiltration of cells in the CNS, mice were killed and the infiltrating cells were enriched from the spinal cords. The results show that EAE-bearing mice recipient of Treg cells had significantly lower infiltrating cells than mice from the EAE and Teff-EAE groups (Fig 6B).

### Violacein-elicited regulatory T cells are endowed with a more robust suppressive activity.

**Fig 7:**
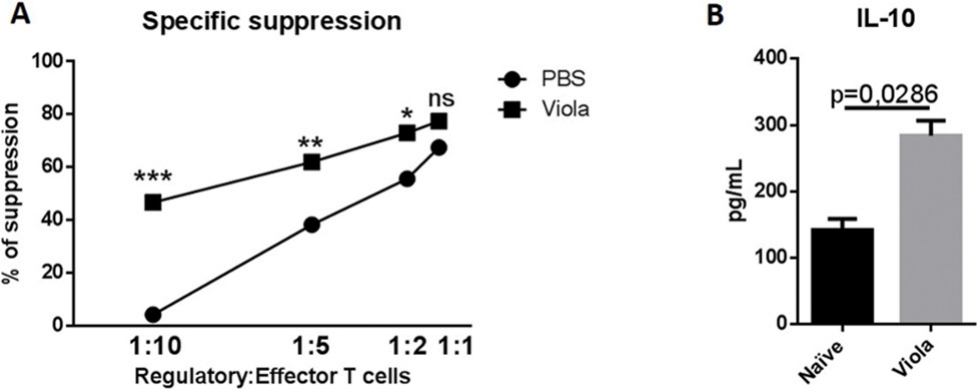
Violacein-elicited regulatory T cells present an enhanced suppressive activity than “naïve” Treg cells. C57BL/6 mice (n = 6 mice/group) were treated with viola (3.5mg/Kg) for three consecutive days. A) After the last dose of viola, mice were killed and spleen cells were prepared for single cell suspension. CD4^+^CD25^+^ and CD4^+^CD25^-^ cells were isolated using dynabeads, following manufacturer’s instructions (Life Technologies). As controls, cells were isolated from spleens from naïve mice. Treg cells were seeded in U-bottom 96-wells culture plate in increasing numbers. C57BL/6 mice (n = 3) were immunized with MOG peptide. After seven days, mice were killed and the spleens were collected and disrupted for the isolation of dendritic cells and total T cells with dynabeads. T cells were stained with CFSE (1,5 μM) and seeded to the plates containing Treg cells (5x10^5^ cells/well). Dendritic cells were isolated as well and seeded to the wells (5x10^4^ DCs/ well). As controls, encephalitogenic T cells were cultivated without Treg cells. The plates were incubated for 72h at 37°C and the suppressive activity of Treg cells was analyzed by flow cytometry. B) Treg cells were isolated from PBS- and viola-treated mice, seeded to 96-well plate and incubated for 48h at 37 ºC. The supernatants were collected and assayed for the detection of IL-10 by CBA. Representative data from two independent experiments. All values are represented as means ± standard error mean. *: p<0.05, **: p<0,01 and ***: p<0,005. Ns: not significant.

We observed that viola treatment augmented the frequency of regulatory T cells in the spleens of mice. It is important to investigate whether these expanded Treg cells retain their suppressive ability compared with Treg cells from naïve mice. For that matter, we performed an experiment in which we compared the suppressive ability of naïve or violacein-derived Treg cells. Splenic Treg cells were isolated from PBS- and viola-treated mice and co-cultivated, at several proportions, with CFSE-stained T cells from MOG-immunized mice for 96h. The suppression ability of naïve and viola-treated Treg cells was evaluated through the analysis of the proliferation of the encephalitogenic T cells. We observed that Treg cells from Viola-treated mice were endowed with a more efficient suppressive ability than cells from PBS-treated mice (Fig 7A). Viola-elicited Treg cells do not express higher levels of CTLA-4 than naïve-derived Treg cells (Fig 4C). However, we found that viola-elicited Treg cells produce significantly higher levels of IL-10 than naïve Treg cells, which suggests that immunosuppression in this model is based on cytokine secretion rather than cell contact (Fig 7B). Still, we did not check for the expression of GITR, PD-L1 and FAS-L on Treg cells. Our group will conduct further studies in order to characterize the mechanism of action of violacein-elicited Treg cells.

## Discussion

In this paper, we show that the quorum sensing metabolite violacein is able to suppress autoimmune neuro-inflammation. Using the Experimental Autoimmune Encephalomyelitis model of human multiple sclerosis and an acute inflammation model, we found that the administration of viola reduced the inflammation through the down-modulation of inflammatory mediators in the Central Nervous System as well as the systemic release of inflammatory cytokines. To our knowledge, we are the first group to present evidence that viola possess anti-inflammatory activity.

Drugs that change the profile of cytokine secretion upon an inflammatory trigger are desired. In this context, the modulation of autoimmune diseases is a common goal in immunology research. In this study, observed that following LPS injection, the treatment with viola was able to reduce the systemic levels of IL-6 while increasing the levels of IL-10. Further analyses showed that the cellular subpopulations of DC, B and T cells were unaltered. Although, viola seems to have a toxic effect on macrophages, suggesting that other subpopulations are involved in the anti-inflammatory effect exerted by viola. Indeed, we found that violacein treatment reduced the infiltration of neutrophils to the peritoneal cavity and this effect correlated to a lower level of CXCL1 in the serum of viola-treated mice. In this context, it was recently demonstrated that violacein reduces the production of CXCL12 and its interaction with CXCR4 in a model of human breast cancer through the inhibition of matrix metalloproteinase-2/9 activity [20]. In addition to its effect on neutrophil migration, viola treatment was able to enhance the frequency and absolute numbers of regulatory T cells. It is well known that Treg cells are important for the control of inflammation [21–23]. Therapies that increase the circulating numbers of Treg cells have proven to be effective in the control of autoimmune inflammation [11, 24–27]. In this context, antimalarial drugs have proven to be excellent anti-inflammatory agents [15, 28, 29]. It was previously shown that dihydroartemisinin reduced the clinical course of EAE through the inhibition of the mammalian target of rampamycin (mTOR) signaling pathway and increase in Treg numbers [12]. Likewise, we showed previously that, chloroquine, and its analogue primaquine, is able to reduce the severity of Experimental Autoimmune Encephalomyelitis through the elicitation of regulatory T cells [11, 30]. Thus, drugs that rise Treg cells numbers are desired to control autoimmunity [22, 31].

We observed that viola-treated mice presented higher frequency of Treg cells than control mice. These viola-elicited Treg cells were, somehow, involved in the suppression of EAE, as high FOXP3 expression was detected in CNS of viola-treated mice, but in a much lower degree in the other groups.

Interestingly, the gene expression profile in the CNS was significantly changed after the treatment with viola. We observed a reduction in the expression of IL-17 in mice treated with viola, but not in EAE-bearing mice control group. IL-17 was recently recognized as a key player in autoimmune diseases, especially in EAE [10, 32–36]. Although it was shown that, inflammation continues in the CNS despite local production of IL-17 [37], our data indicate that the modulation of EAE by viola is intimately related to the reduced production of IL-17. Of note, IFN-γ expression levels were not changed in the treatment regimen, which indicate that the mechanism of viola-induced suppression is independent of this cytokine in the direct treatment. Notwithstanding, viola-treated mice presented higher expression of IDO and IL-10. Dendritic cells expression of Indoleamine 2,3—Dioxygenase (IDO) enzyme is believed to play a significant role in the suppression of activated effector T cells and induction of regulatory T cells [38–40]. This finding indicates that one of the actions exerted by viola involves the modulation of dendritic cells as well as induction of Treg cells. Further studies will be conducted in order to investigate this possible association.

The role of regulatory T cells in the anti-inflammatory activity of viola was confirmed when we transferred viola-elicited Treg cells towards EAE-bearing mice. Thus, the adoptive transfer was able to overcome the inflammatory process and suppressed the disease even after mice developed the initial symptoms of EAE. Similarly as in chloroquine-elicited Treg cells [11], these transferred regulatory T cells were generated in naïve mice (not in MOG-immunized mice), thus we are persuaded to believe that a bystander suppression was promoted by viola-induced Treg cells. One interesting finding is that, despite no differences in the expression of CTLA-4, violacein-elicited Treg cells were endowed with a more suppressive ability than “naïve” Treg cells, as they controlled the proliferation of encephalitogenic T cells at lower numbers. This may be explained by the higher secretion of IL-10 from viola-elicited Treg cells compared with “naïve” Treg cells.

Other mechanism by which viola may have exerted its effects is the suppression of inflammation *in locu*. It is known that following MOG-immunization, encephalitogenic T cells migrate towards the CNS after 10 days, where they promote inflammation [10, 41, 42]. As the treatment with viola and the adoptive transfer of Treg cells were initiated at this time-point, it is highly probable that part of the anti-inflammatory effect of viola was exerted locally in the CNS. The following observations corroborate this hypothesis: (i) viola-treated mice presented as much as 10x10^5^ cells in the CNS, whereas mice recipient of Treg cells presented 0.2x10^5^ cells and (ii) there were differences in the gene expression between EAE-viola and EAE+CD25^+^ groups, mainly in FOXP3 and IFN-γ expression. Taken together these findings indicate that the adoptively transferred regulatory T cells inhibited the migration of encephalitogenic T cells towards the CNS, whereas the treatment with viola suppressed the inflammatory cells that had already infiltrated the CNS. This hypothesis is supported by the differences between IFN-γ expression in the CNS of EAE bearing mice treated with viola and recipient of Treg cells. Notwithstanding, violacein treatment of naïve mice stimulated the production of IFN-γ and IL-10, but had little effect on IL-17, by T cells. Which may be explained by the fact that violacein is a compound derived from bacteria and therefore may be recognized as such by the immune system. As Th17 cells are responsible for aggravated EAE score, the stimulation of IFN-γ may promote the development of mild EAE [19, 43–45]. Still, further studies must be conducted in order to characterize the mechanism of immune modulation exerted by violacein.

Although viola demonstrated impressive therapeutic potential, this metabolite is toxic at large concentrations. Mice treated with viola at a 7mg/Kg dose succumbed after the second dose of the treatment. In Ehrlich ascites tumors, the treatment with viola induced apoptosis of the tumor cells through oxidative stress and imbalance in the antioxidant defense of cells [5]. A similar mechanism was observed in Caco-2, human colon cancer cells, where reactive oxygen species were induced at high levels leading to cell death [7]. Chloroquine presents similar effects in tumor cells and in lymphocytes [15]. Antimalarials are also known for their toxicity. In this context, we showed that dendritic cells are an interesting approach to bypass this hindrance. When DCs were modulated by chloroquine they acquired an immature phenotype and suppressed EAE upon adoptive transfer [24]. These results suggest that although toxic, some compounds may be used to modulate dendritic cells towards an inflammatory/anti-inflammatory profile to be used as an adjunct therapy. It is possible that viola-modulated dendritic cells acquire an immature phenotype as well as chloroquine-modulated DCs. Further studies must be conducted to ascertain this possibility.

## Conclusion

In this study, we show that violacein, a quorum sensing metabolite, has anti-inflammatory activity through the stimulation of regulatory T cells and local suppression of inflammation. Viola-elicited Treg cells were also more suppressive than Treg cells isolated from naïve mice. Although violacein is toxic at high doses, future studies will be conducted in order to overcome this toxicity through the usage of violacein-modulated dendritic cells.
